# Lack of association of the *KIR* and HLA class I ligands with ZIKV infection in south and southeast of Brazil

**DOI:** 10.1590/0074-02760210194

**Published:** 2022-08-15

**Authors:** Laise Nayana Sala Elpidio, Amarilis Giaretta de Moraes, Ieda Bernadete Volkweis Langer, Greicy Cezar do Amaral, Maria Luiza Moretti, Márcia Teixeira Garcia, Rodrigo Angerami, José Luiz Proenca-Modena, Karina Bispo-dos-Santos, Matheus Cavalheiro Martini, Pierina Lorencini Parise, Christiane Maria Ayo, Luiz Carlos de Mattos, Cinara Cássia Brandão, Maurício Lacerda Nogueira, Denise Cristina Mós Vaz Oliani, Lígia Cosentino Junqueira Franco Spegiorin, Quirino Alves de Lima Neto, Jeane Eliete Laguila Visentainer

**Affiliations:** 1 Universidade Estadual de Maringá, Programa de Pós-Graduação em Biociências e Fisiopatologia, Departamento de Análises Clínicas e Biomedicina, Maringá, PR, Brasil; 2 Farmacêutica responsável pelo Laboratório da Unidade Central de Nova Prata do Iguaçu, Nova Prata do Iguaçu, PR, Brasil; 3 15ª Regional de Saúde do Paraná, Maringá, PR, Brasil; 4 Universidade Estadual de Campinas, Faculdade de Ciências Médicas, Departamento de Medicina Interna, Campinas, SP, Brasil; 5 Universidade Estadual de Campinas, Divisão de Epidemiologia Hospitalar, Hospital das Clínicas, Campinas, SP, Brasil; 6 Departamento de Vigilância em Saúde Pública de Campinas, Campinas, SP, Brasil; 7 Universidade Estadual de Campinas, Instituto de Biologia, Departamento de Genética, Microbiologia e Imunologia, Laboratório de Vírus Emergentes, Campinas, SP, Brasil; 8 Faculdade de Medicina de São José do Rio Preto, Departamento de Biologia Molecular, Laboratório de Imunogenética, São José do Rio Preto, SP, Brasil; 9 Faculdade de Medicina de São José do Rio Preto, Departamento de Doenças Infecciosas e Parasitárias, Laboratório de Pesquisa em Virologia, São José do Rio Preto, SP, Brasil; 10 Faculdade de Medicina de São José do Rio Preto, Departamento de Ginecologia e Obstetrícia, São José do Rio Preto, SP, Brasil; 11 Universidade Estadual de Maringá, Laboratório de Imunogenética, Maringá, PR, Brasil

**Keywords:** genetic association studies, HLA class I, KIR receptors, natural killer cell, Zika virus infection

## Abstract

**BACKGROUND:**

Zika virus (ZIKV) is an emerging arbovirus associated with foetal malformations and neurological complications. The infection is usually associated with mild symptoms. The comparison between the allelic frequency of polymorphic genes in symptomatic infected individuals in the population can clarify the pathogenic mechanisms of ZIKV. During ZIKV infection, cytokines are produced and natural killer (NK) cells are recruited, whose activation depends on signaling pathways activated by specific receptors, such as killer cell immunoglobulin-like receptors (KIR). These molecules interact with human leukocyte antigen (HLA) class I ligands and are encoded by polymorphic genes.

**OBJECTIVES:**

This study aimed to evaluate the frequency of allelic variants of the genes encoding the KIR receptors and their *HLA* class I ligands in 139 symptomatic ZIKV-patients and 170 controls negative for the virus, and to evaluate the role of these variants for ZIKV susceptibility.

**METHODS:**

*KIR* and *HLA* class I genes were genotyped using the polymerase chain reaction-sequence specific oligonucleotide (PCR-SSO) technique.

**FINDINGS:**

No significant differences in the frequency distribution of *KIRs* and KIR-HLA in patients compared to controls were observed.

**MAIN CONCLUSIONS:**

KIR and its HLA ligands might play a minor role in ZIKV infection in the south and southeast Brazilian individuals.

The Zika virus (ZIKV) is a flavivirus that mostly causes asymptomatic or self-limited symptoms.^1^ Symptoms generally include fever, conjunctivitis, rash, myalgia, arthralgia, malaise, and headache, and usually last for two-seven days.^2^ In 2016, ZIKV infection was recognised as a global emerging and public health problem due to the upsurge of evidences of severe illnesses like Guillain-Barré syndrome (GBS), neurological abnormalities and foetal malformations.^3^ ^,^ ^4^

Between 2015-2016, Brazil experienced an epidemic of the virus with 1,673,272 suspected cases of ZIKV infection and 1,950 confirmed cases of microcephaly from ZIKV infection.^5^ Currently, even with the reduction in cases, in 2020 more than 7,000 probable cases of ZIKV infection were reported and a new strain of the virus with the potential to trigger a second epidemic was discovered in Brazil.^6^ ^,^ ^7^

The innate immune response is essential for the control of flavivirus infection and involves the recruitment of innate immune cells, such as natural killer (NK) cells.^8^ NK cells express receptors that modulate their function. The activating and inhibitory Killer cell immunoglobulin-like receptors (KIR) are the main receptors involved in controlling the NK response.^9^ These molecules interact with human leukocyte antigen (HLA) class I ligands expressed by target cells.^9^ ^,^ ^10^ Thus, the signals resulting from the KIR-HLA class I interaction modulate the activation or inhibition of the effector function of NK cells.^9^ ^,^ ^10^

The genes on chromosome 19q13.4 region encode the KIR protein.^11^ There are fifteen functional genes, classified as inhibitors (*KIR2DL* and -*3DL*) and activators (*KIR2DS* and -*3DS*) of NK cells, and two pseudogenes (*KIR2DP1* and -*3DP1*).^12^ *KIR2DS4* (*KIR2DS24-Del* variant) has a 22bp deletion in exon 5, which results in a soluble molecule with loss of the cytoplasmic domain. In contrast, *KIR2SDS4-full* is free from deletion and encodes a molecule that is capable of anchoring itself on the NK surface.^13^ The genes that encode HLA class I molecules, ligands of KIR, are located at the 6p21.3 chromosomal region.^14^

The *KIR* genes and their HLA class I ligands are associated with diseases caused by flaviviruses, such as dengue and West Nile viruses.^15^ ^,^ ^16^ ^,^ ^17^ ^,^ ^18^ ^,^ ^19^ To date, the influence of *KIR* genes and their HLA class I ligands on patients affected by ZIKV has not been evaluated yet. By investigating the KIR-HLA association, we might better understand the genetic susceptibility involved in ZIKV infection. In this sense, the objective of this study was to evaluate the possible association of the genes encoding the KIR receptors and their HLA class I ligands in susceptibility or resistance to Zika virus infection.

## SUBJECTS AND METHODS

The study was carried out according to the Human Research Ethics Committees of the State University of Maringá and State University of Campinas (CAAE 2.364.256/2017), and the Medical School of São José do Rio Preto (CAAE 55805516.2.0000.5415).

*Patients and controls* - The studied population was formed by individuals who sought medical care during the ZIKV outbreak (2015-2017), in the regions of Ma-ringá (Paraná) and São Paulo (Campinas and São José do Rio Preto), in Brazil, presenting with clinical manifestations of the disease, such as fever, headache, exanthema, among others. Individuals who had ZIKV clinical and laboratory aspects, evaluated by experienced doctors, were invited to participate in the study. Following the recommendations of the World Health Organization (WHO), suspected individuals consisted of those with skin rash and/or fever and at least one of the following clinical manifestations: arthralgia or arthritis or conjunctivitis.^20^ A total of 309 individuals were included in this study, and two groups were formed, as follows. The control group consisted of 170 non-related individuals with ZIKV-like symptoms but tested negative for ZIKV. These subjects also tested negative for dengue virus (DENV) and/or co-circulating arboviruses due to the possible cross-reactivity. The case group consisted of 139 individuals presenting two or more clinical manifestations of ZIKV, according to WHO and tested positive for ZIKV laboratory tests.

All participants of this study were over 18 years of age. Inclusion criteria included individuals who had a clinical evaluation, and laboratory diagnosis and were not related.

Of the total participants (N = 309), 81 individuals (47 patients and 34 controls) were residents of the state of Paraná, Brazil. Also, 101 DNA samples (from 52 patients and 49 controls) were kindly provided by the Laboratory for the Study of Emerging Viruses (LEVE) of the Institute of Biology at the University of Campinas - Campinas, São Paulo. One hundred and twenty-seven DNA samples (from 40 patients and 87 controls) were kindly provided by the Laboratory of Immunogenetics of the Faculty of Medicine of São José do Rio Preto - São José do Rio Preto, São Paulo, Brazil.

Study participants were considered miscegenated due to the high degree of heterogeneity of the Brazilian population, whose Caucasian population is predominantly European (80.6%), African (12.5%) and Amerindian (7.0%).^21^

*Detection of the ZIKV* - The detection of the ZIKV in samples from Paraná was performed using the real-time quantitative reverse transcription-polymerase chain reaction (RT-qPCR) technique executed by the Central Laboratory of Paraná (LACEN), Laboratory of Teaching and Research and Clinical Analysis of the State University of Maringá (LEPAC) and the Oswaldo Cruz Foundation (Fiocruz). Samples provided by the partner laboratories of Campinas and São José do Rio Preto, ZIKV detection was performed in blood and urine specimens using the QuantiNova Probe RT-PCR kit (Qiagen, Hilden, Germany), according to Judice et al.,^22^ and by Lanciotti et al.^23^

Individuals who tested negative for ZIKV by the PCR method were also tested for DENV or other arboviruses. Samples from Paraná were submitted to a Real-time RT-PCR to test for arboviruses co-circulating with ZIKV at that time, which included the four DENV serotypes and Chikungunya virus (CHIKV) and/or DENV detection by IgM antibodies was performed. Samples from São Paulo were tested for DENV using rapid tests specific for NS1 antigen and IgM antibodies. Based on epidemiological data during the ZIKV epidemic in Brazil, CHIKV, Oropouche and Mayaro were not co-circulating at that time and therefore tests were not performed on those subjects. In samples of man and non-pregnant women from São José do Rio Preto, the IgG enzyme-linked immunosorbent assay (ELISA) kit for human anti-dengue virus (Abcam, Cambridge, UK) and ZIKA-v IgG kit (Advangen Biotech, Itu-São Paulo, Brazil) were used. The ZIKA-v IgG Kit was used to measure the responses of the immunoglobulin G3 (IgG3) to the ZIKV NS1 antigen. When the ZIKV IgG antibody testing by ELISA methodology was positive, a confirmatory plaque reduction neutralisation test (PRNT) was performed against Zika.

*Sample collection and processing* - For individuals from the Paraná region, who agreed to participate in the research, approximately 5 mL of venous blood were collected. DNA extraction was performed using the QIAamp^®^ DNA Blood Mini Kit (Qiagen) and Biopur^®^ (Mobius, Brazil) kits. DNA was quantified in the NanoDrop2000^®^ equipment (Wilmington, USA). Blood samples from Campinas and São José do Rio Preto were obtained at the time of diagnosis and DNA was extracted by QIAamp^®^ DNA Blood Mini Kit (Qiagen) in the laboratory of origin. DNA samples and spreadsheets containing clinical and laboratory results were sent to the Laboratory of Immunogenetics of the State University of Maringá (LIG-UEM), Brazil.

*Genotyping of KIR and HLA class I* - Genotyping of the *KIR* and *HLA-A*, *HLA-B* and *HLA-C* genes were performed using the polymerase chain reaction-sequence specific oligonucleotides (PCR-SSO) technique with the kits LABType KIR SSO Genotyping Test and LABType SSO (One Lambda Inc., Canoga Park, CA, USA) according to the manufacture’s instruction. The genotyping kit for the 16 *KIRs* genes contains specific primers for exons 3, 4, 5, 7, 8 and 9 and allows the identification of the presence and absence of the gene. The genotyping kit for the *HLA-A*, *HLA-B* and *HLA-C* genes has specific primers for exons 2-3 and allows for medium resolution and high definition. In this technique, the amplified DNA is hybridised to probes that are connected to fluorescent microspheres and its detection is performed by the Conjugated Streptavidin /Phycoerythrin reagent (SAPE). Hybridisation was verified by a flow cytometry (LABScan TM 100 flow analyser), which detects fluorescence intensity. The results were interpreted using HLA FUSION 4.2 software (One Lambda).

The HLA-KIR ligands specificities were considered as follow: HLA molecules from the C1 group (HLA-C*01, *03*, *07, *08, *12, *14, *16) are ligands of KIR2DL2, 2DL3, and 2DS2.^9^ ^,^ ^24^ ^,^ ^25^ ^,^ ^26^ HLA molecules from the C2 group (HLA-C*02, *04, *05, *06, *15, *17, *18) are ligands of KIR2DL1 and KIR2DS1.^24^ ^,^ ^25^ ^,^ ^26^ Bw4 epitopes (HLA-A*23, A*24, A*25, A*32, B*13, B*27, B*37, B*38, B*44, B*49, B*51, B*52, B*53, B*57, B*58), Bw4-80Ile (Bw4 with isoleucine at position 80: HLA-A*23, *24, *25, *32, *15:17, *27:02, *38:01, *49:01, *51, *52:01, *53:01, *57, *58) and Bw4-80Thr (Bw4 with threonine at position 80: HLA-B*13, *27:05, *37:01, *44) are recognised by KIR3DL1.^27^ ^,^ ^28^ ^,^ ^29^ The specificities HLA-A*03 and A*11 are ligands of KIR3DL2.^25^ HLA-C*04 is ligand for KIR2DS4, which has been subdivided into *2DS4-full* (*003, *004, *006, *007, *008, *009, *010, *012, *013) and *2DS4-del* (*001*, *011, *014, *015), as previously described.^13^ ^,^ ^30^

The Allele Frequency Net Database was used to obtain the ID genotype and AA and Bx groups haplotypes (http://www.allelefrequencies.net/kir6001a.asp). The *KIR* genes were grouped into two haplotype groups based upon gene content and designated as AA and Bx: The combination of *3DL3*, *2DL3*, *2DP1*, *2DL1*, *3DP1*, *2DL4*, *3DL1*, *2DS4* and *3DL2* genes and the absence of any additional *KIR* gene composed the AA haplotype group. The Bx haplotype group are characterised by the presence of one or more of the following genes: *KIR2DS2*, *2DL2*, *3DS1*, *2DL5*, *2DS3*, *2DS5* and *2DS1*. They may also have, as often happens, *KIR* genes belonging to the AA haplotype group.

*KIR2DL2* and *KIR2DL3* as well as *KIR3DL1* and *KIR3DS1* segregate as alleles of the same locus. For these pairs of genes, the distribution of the homozygous and heterozygous genotypes was calculated.^31^

*Statistical analysis* - The frequencies of the *KIR* genes and KIR-HLA class I ligand was obtained by direct counting. The comparison of gene frequencies between patients and controls was performed using the Chi-square test with Yates correction or Fisher’s exact test using the 2x2 contingency table of the Open Epi software, available at https://www.openepi.com /Menu/OE_Menu.htm. The chance of association was evaluated by calculating the odds ratio (OR) considering the 95% confidence interval (95% CI). P < 0.05 was considered significant.

The Hardy-Weinberg equilibrium (HWE) was obtained for *KIR2DL2/3*, *KIR3DL1/S1* and HLA class I using the software Arlequin 3.5.1.3 (available at http://en.bio-soft.net/other/arlequin.html) after organising the data in Convert software 1.31.^32^ ^,^ ^33^ ^,^ ^34^ The statistical power was calculated using GPower 3.1. software, and considering our sample size, the prevalence of the less frequent *KIR* gene (28%) and OR = 2, the statistical power was considered sufficient (> 0.80).^35^ The multiple comparisons correction with adjustment for gender and age covariates was performed by logistic regression analysis using the R software, version 4.1.2, with the stats and gtsummary packages (available at: https://www.r-project.org/).

## RESULTS

Of the 309 individuals enrolled in this study, 139 (117 women and 22 men, mean age 35.9 ± 14.38 years) composed the case group. Table I shows the main clinical manifestations reported by the patients. The lack of equal and complete clinical information in medical records, together with its electronic transformation, made it difficult for us to access data on the clinical manifestations of all patients included in our study. Therefore, we obtained access to the clinical manifestations of 98 individuals (Table I). The control group consisted of 170 individuals (88 women and 82 men, mean age 42.2 ± 15.36). The group of patients presented a significantly lower mean age compared with the group of control individuals (p = 0.0003) (Table I). Differences regarding sex were also observed between the groups: the ZIKV-infected group was composed mostly of women compared to the control group (p < 0.0001) (Table I).

**TABLE I:** Sociodemographic data and main clinical manifestations reported by patients affected by Zika virus (ZIKV)

Characteristics	Patients	Controls	p-value
Age	35.9 (± 14.38)	42.2 (± 15.36)	0.0003
Gender			
Women	117	88	p < 0.0001
Men	22	82	p < 0.0001
	Patients (N = 98)		
Clinical manifestations	n	Frequency (%)	
Exanthema (rash)	89	91	
Body pain and/or muscle/joint pain	52	53	
Pruritus	46	47	
Headache	46	47	
Fever	44	45	
Retroorbital and/or red/irritated eyes	18	18	
Somnolence and weakness	16	16	

Note: values shown as mean ± standard deviation.

*KIR* (*KIR2DL2/3* and *KIR3DL1/S1*) and *HLA* class I frequencies were in HWE (p-value > 0.05) and the frequency of distribution of the *KIR* genes in the control group were in agreement with those observed in other healthy populations of the same geographical region.^36^ ^,^ ^37^ Also, all participants of this study have the respective framework genes *KIR2DL4*, *KIR3DL2*, *KIR3DL3*, and *KIR3DP1*. Therefore, validating ZIKV-negative individuals as a good control group for this study.

No significant differences were found in the distribution of *KIR* genes and of homozygous and heterozygous genotypes of *KIR2DL2/KIR2DL3* and *KIR3DL1/KIR3DS1* between the groups (Table II).

**TABLE II:** Frequency distribution of the presence of the *KIR* genes and genotypes in Zika virus (ZIKV) patients and controls

	Patients (N = 139)		Controls (N = 170)		
*KIR* genes	n	Frequency (%)	n	Frequency (%)	p-value
Inhibitory					
*2DL1*	130	93.5	161	94.7	0.8441
*2DL2*	78	56.1	90	52.9	0.6582
*2DL3*	119	85.6	150	88.2	0.6078
*2DL5*	74	53.2	89	52.4	0.9678
*3DL1*	134	96.4	159	93.5	0.3810
Activating					
*2DS1*	53	38.1	69	40.6	0.7468
*2DS2*	79	56.8	90	52.9	0.5693
*2DS3*	41	29.5	48	28.2	0.9066
*2DS5*	51	36.7	62	36.5	0.9372
*3DS1*	51	36.7	66	38.8	0.7897
*2DS4*	134	96.4	159	93.5	0.3810
Full	101	72.7	126	74.1	0.8738
Del	108	77.7	125	73.5	0.4754
Full/Del	75	54.0	92	54.1	0.9311
Framework and pseudogenes					
*2DL4*	139	100.0	170	100.0	> 0.9999
*3DL2*	139	100.0	170	100.0	> 0.9999
*3DL3*	139	100.0	170	100.0	> 0.9999
*3DP1*	139	100.0	170	100.0	> 0.9999
*2DP1*	133	95.7	161	94.7	0.8952
*KIR* genotypes	n	%	n	%	
*KIR3DL1/KIR3DS1*	46	33.1	55	32.4	0.9871
*KIR3DL1/KIR3DL1*	88	63.3	104	61.2	0.7897
*KIR3DS1/KIR3DS1*	5	3.6	11	6.5	0.3810
*KIR2DL2/KIR2DL3*	58	41.7	70	41.2	0.9853
*KIR2DL2/KIR2DL2*	20	14.4	20	11.8	0.6078
*KIR2DL3/KIR2DL3*	61	43.9	80	47.1	0.6582

ns: non-significant results; n: number of individuals; *KIR2DS4-full* (*003, *004, *006, *007, *008, *009, *010, *012, *013); *KIR2DS4-del* (*001, *011, *014, *015).

Thirty-seven different haplotypes (AA and Bx) of the *KIR* gene were identified [Supplementary data (Table I)]. However, there was no statistically significant difference (p > 0.05) in the frequency distribution of the haplotype groups when patients and controls were compared.

Also, no significant differences were found in the distribution of frequencies of HLA class I ligands of KIR (HLA-A*03 or HLA-A*11, HLA-Bw4, HLA-Bw4-Ile, HLA-Bw4-Thr, HLA-C1, and HLA-C2) and in the distribution of KIR-HLA receptor-ligand pairs between the groups (Tables III-IV, respectively).

**TABLE III:** Frequencies of human leukocyte antigen (HLA) class I ligands in Zika virus (ZIKV) patients and controls

	Patients (N = 139)		Controls (N = 170)		
HLA CLASS I ligands	n	Frequency (%)	n	Frequency (%)	p-value
A*03	26	18.7	29	17.1	0.8205
A*11	19	13.7	16	9.4	0.3201
A*03 and/or A*11	43	30.9	43	25.3	0.3305
Bw4	112	80.6	142	83.5	0.5990
Bw4/Bw4	46	33.1	51	30.0	0.6457
Bw4- 80Ile	84	60.4	119	70.0	0.1006
Bw4-80Ile/ Bw4-80Ile	27	19.4	32	18.8	0.9906
Bw4-80Thr	43	30.9	50	29.4	0.8683
Bw4-80Thr/80Thr	5	3.6	2	1.2	0.2999
C*04	47	33.8	51	30.0	0.5528
C1	110	79.1	134	78.8	0.9417
C2	95	68.3	117	68.8	0.9736
C1/C2	66	47.5	81	47.6	0.9318
C1/C1	44	31.7	53	31.2	0.9736
C2/C2	29	20.9	36	21.2	0.9417

n: number of individuals; ns: non-significant results; ^#^The same individual could express more than one pair of HLA ligands. Bw4: HLA-A*23, A*24, A*25, A*32, B*13, B*27, B*37, B*38, B*44, B*49, B*51, B*52, B*53, B*57, B*58; Bw4-80Ile: HLA-A*23, *24, *25, *32, *15:17, *27:02, *38:01, *49:01, *51, *52:01, *53:01, *57, *58; Bw4-80Thr: HLA-B*13, *27:05, *37:01, *44; Group C1: HLA-C*01, *03*, *07, *08, *12, *14, *16; Group C2: HLA-C*02, *04, *05, *06, *15, *17, *18.

**TABLE IV:** Comparison of the frequencies of *KIR* genes in the presence of their respective human leukocyte antigen (HLA) class I ligands between Zika virus (ZIKV) patients and controls^*a*^

	Patients (N = 139)		Controls (N = 170)		
KIR-HLA CLASS I ligands pair	n	Frequency (%)	n	Frequency (%)	p-value
2DL1-C2	88	63.3	113	66.5	0.6456
2DL1-C2/C2	28	20.1	33	19.4	0.9863
2DL1-C1/C2	60	43.2	80	47.1	0.5693
2DL2-C1	63	45.3	69	40.6	0.4706
2DL2-C1/C1	24	17.3	28	16.5	0.9736
2DL2-C1/C2	39	28.1	34	20.0	0.1275
2DL3-C1	94	67.6	119	70.0	0.7451
2DL3-C1/C1	39	28.1	44	25.9	0.7641
2DL3-C1/C2	55	39.6	75	44.1	0.4902
3DL2-A*03/A*11	43	30.9	43	25.3	0.3305
3DL2-A*03/A*11(homo)	6	4.3	2	1.2	0.1710
3DL1-Bw4	106	76.3	136	80.0	0.5124
3DL1- Bw4-80Ile	80	57.6	111	65.3	0.2025
3DL1- Bw4-80Ile/ 80Ile	25	18.0	30	17.6	0.9425
3DL1- Bw4-80Thr	41	29.5	45	26.5	0.6435
3DL1- Bw4-80Thr/80Thr	5	3.6	2	1.2	0.2999
3DL1-Bw4/Bw4	44	31.7	47	27.6	0.5199
2DS1-C2	36	25.9	52	30.6	0.4343
2DS1-C2/C2	9	6.5	17	10.0	0.3657
2DS1-C1/C2	27	19.4	35	20.6	0.9113
2DS2-C1	60	43.2	71	41.8	0.8948
2DS2-C1/C1	25	18.0	28	16.5	0.8417
2DS2-C1/C2	38	27.3	43	25.3	0.7822
2DS4-C*04	46	33.1	48	28.2	0.4242
2DS4-C*04/C*04	5	3.6	6	3.5	0.7821
2DL2/2DL2-C1	16	11.5	15	8.8	0.5539
2DL2/2DL3-C1	47	33.8	54	31.8	0.7949
2DL3/2DL3-C1	47	33.8	65	38.2	0.4930
2DL2/2DL2-C1/C2	11	7.9	6	3.5	0.1526
2DL2/2DL3-C1/C2	28	20.1	35	20.6	0.9637
2DL3/2DL3-C1/C2	27	19.4	40	23.5	0.4639
2DL2/2DL2-C1/C1	5	3.6	9	5.3	0.6609
2DL2/2DL3-C1/C1	19	13.7	19	11.2	0.6244
2DL3/2DL3-C1/C1	20	14.4	25	14.7	0.9335
2DS2/2DL2-C1	60	43.2	68	40.0	0.6557
2DS2/2DL2-C1/C1	24	17.3	27	15.9	0.8635
2DS2/2DL2-C1/C2	36	25.9	41	24.1	0.8196

*a*: non-significant differences; n: number of individuals; Group C1: HLA-C*01, *03, *07, *08, *12, *14, *16 ligands for KIR2DL2, 2DL3 and 2DS2; Group C2: HLA-C*02, *04, *05, *06, *15, *17, *18 ligands for KIR2DL1 and KIR2DS1; Bw4: HLA-*A23, A*24, A*25, A*32, B*13, B*27, B*37, B*38, B*44, B*49, B*51, B*52, B*53, B*57, B*58 ligands for KIR3DL1 and KIR3DS1; Bw4-80Ile: HLA-A*23, *24, *25, *32, *15:17, *27:02, *38:01, *49:01, *51, *52:01, *53:01, *57, *58 ligands for KIR3DL1 and KIR3DS1; Bw4-80Thr: HLA-B *13, *27:05, *37:01, *44 ligands for KIR3DL1 and KIR3DS1; HLA-A*03 and *11 ligands for KIR3DL2; HLA-C*04 ligands for KIR2DS4.

Analysis employing logistic regression with adjustment for age and sex indicated no association between *KIR* and KIR-ligand class I when patients were compared to controls [Supplementary data (Table II)].

## DISCUSSION

According to our knowledge, this is the first study to assess the influence of *KIR* and HLA class I ligands on ZIKV infection. KIR-HLA genetic variability influences the ability to generate signals to the NK cell and can intervene in the response of these cells to infections.^9^ ^,^ ^11^ ^,^ ^38^ The expression of a specific KIR, HLA class I ligand or both may be absent and result in a deficiency of NK function.^26^ In this sense, the genetic profile of each individual combined with the polymorphisms present in each *KIR* gene gives KIR receptors the characteristic of being diverse^9^ and might be associated with diseases.^26^

It is worth mentioning that ZIKV infection can cause positive expression of HLA class I molecules.^39^ These proteins can interact with inhibitory KIRs and, consequently, ZIKV supposedly evades the response of NK cells.^39^ However, the results obtained in this study showed no association between the genes encoding the KIR receptors and their HLA ligands with ZIKV infection. Despite few studies showing an association of *KIR* genes with other flaviviruses, Beltrame et al.^15^ and Ramanathan et al.^18^ suggested a possible influence of the *KIR2DL5*, *KIR2DL2* and *KIR3DL1* genes on susceptibility to dengue infection. It has been proposed that at the beginning of the dengue infection, there is an increase in the presentation of NS1 by HLA-B27, expressed on the surface of the infected cell, and the interaction of the NS1-HLA-B27 complex with KIR3DL1 causes the inhibition of NK.^40^ In patients with dengue, Chaisri et al.^19^ indicated susceptibility of HLA-A*11 in the development of the disease. In addition to the interaction with KIRs, studies indicate that HLA-A*24 and HLA-B*44, variants of the HLA-Bw4 ligand, with the respective T cell responses are related to susceptibility to dengue hemorrhagic fever.^41^ ^,^ ^42^

Zika displayed uneven epidemiological outcomes across Brazil. Whereas the Northeast region was severely hit by the 2016 ZIKV epidemic, in the Southern, < 0.5% of ZIKV infections had been reported in the same period.^43^ Furthermore, ZIKV infection can affect both sexes at any time in life. However, based on our study and the literature, it is important to emphasise that women were reported most affected by infection^44^ ^,^ ^45^ ^,^ ^46^ and the predominant age group of ZIKV cases in women was 20 to 39 years.^46^ Additionally**,** we chose to include individuals with laboratory diagnoses of ZIKV infection in the research. In this sense, the Pan American Health Organization (PAHO) recommends, except for pregnant women, that laboratory tests should be performed on individuals considered to be suspected, that is, individuals symptomatic for ZIKV infection.^47^ Because of this, we chose to include symptomatic individuals with a negative diagnosis for ZIKV in the control group.

Other factors may have been the causes of the clinical manifestations presented by the control group. Among them, we can mention infections by respiratory viruses, such as influenza, urinary infections and other arboviruses. We emphasise that all care was taken with the collection of biological samples and techniques performed for laboratory diagnosis that minimised the possibility of false-negative results for ZIKV infection.

Due to the low number of individuals with the severe form of the disease in the study region, it was not possible to assess the contribution of the *KIR* and HLA class I genes in the different forms of ZIKV infection. In addition, the evaluation of these genes in different regions of Brazil and other countries that have faced ZIKV epidemics may provide more information about the pathophysiology of ZIKV infection. Additionally, to better elucidate the role of KIR receptors in ZIKV infection, it would be necessary to carry out further studies involving the allelic polymorphisms of *KIR* genes, expression of these receptors and cytotoxicity assays of NK cells.

In short, there was no evidence of an association between the genes encoding the KIR receptors and their HLA class I ligands in ZIKV infection in the south and southeast Brazilian individuals.
