# Supplementary material for: Lack of association of the KIR and HLA class I ligands with ZIKV infection in south and southeast of Brazil
Source: Mem Inst Oswaldo Cruz. 2022 Aug 15;117:e210194. doi: 10.1590/0074-02760210194 (PMC9377541; doi:10.1590/0074-02760210194)
Supplement: Supplementary file 1 [file 1678-8060-mioc-117-e210194-s.pdf]

TABLE I

Frequencies of *KIR* haplotype groups in Zika virus (ZIKV) patients (N = 139) and controls (N = 170)

Genotype group	ID genotype	2DL1	2DL2	2DL3	2DS1	2DS2	2DS3	2DS4	2DS5	2DL4	3DL1	3DL2	3DS1	3DL3	2DL5	2DP1	3DP1	Patients		Controls		p-value[1]	p-value[2]
																		n	%	n	%		
AA	1	■	□	■	□	□	□	■	□	■	■	■	□	■	□	■	■	38	27.3	50	29.4	0.7832	0.5
Bx	2	■	□	■	■	□	□	■	■	■	■	■	■	■	■	■	■	15	10.8	21	12.4	0.8046	0.2
Bx	4	■	■	■	□	■	□	■	□	■	■	■	□	■	□	■	■	20	14.4	21	12.4	0.7217	0.12
Bx	5	■	■	■	□	■	■	■	□	■	■	■	□	■	■	■	■	10	7.2	15	8.8	0.7544	0.8
Bx	3	■	■	■	■	■	□	■	■	■	■	■	■	■	■	■	■	6	4.3	7	4.1	0.8429	0.13
Bx	6	■	■	■	■	■	■	■	■	■	■	■	■	■	■	■	■	8	5.8	9	5.3	0.9411	0.6
Bx	9	■	■	■	■	■	□	■	■	■	■	■	□	■	■	■	■	0	0.0	2	1.2	> 0.9999	-
Bx	7	■	■	■	■	■	■	■	□	■	■	■	■	■	■	■	■	6	4.3	5	2.9	0.7335	0.3
Bx	90	■	■	□	■	■	■	■	□	■	■	■	■	■	■	■	■	0	0.0	1	0.6	> 0.9999	-
Bx	71	■	■	□	□	■	■	■	□	■	■	■	□	■	■	■	■	5	3.6	1	0.6	0.1343	0.2
Bx	72	□	■	□	□	■	□	■	□	■	■	■	□	■	□	□	■	4	2.9	6	3.5	> 0.9999	0.8
Bx	73	■	■	□	■	■	■	■	■	■	■	■	■	■	■	■	■	3	2.2	3	1.8	> 0.9999	0.6
Bx	70	■	■	■	■	■	■	□	■	■	□	■	■	■	■	■	■	1	0.7	4	2.4	0.5090	0.14
Bx	8	■	□	■	■	□	■	■	□	■	■	■	■	■	■	■	■	2	1.4	2	1.2	> 0.9999	0.8
Bx	68	■	■	■	■	■	□	□	■	■	□	■	■	■	■	■	■	1	0.7	1	0.6	> 0.9999	0.7
Bx	69	■	□	■	■	□	□	□	■	■	□	■	■	■	■	■	■	0	0.0	3	1.8	0.7806	-
Bx	76	□	■	□	■	■	□	■	■	■	■	■	■	■	■	□	■	1	0.7	3	1.8	0.7806	-
Bx	81	■	■	□	■	■	■	□	■	■	□	■	■	■	■	■	■	1	0.7	3	1.8	0.7806	0.6
Bx	19	■	■	■	□	□	□	■	□	■	■	■	□	■	□	■	■	1	0.7	1	0.6	> 0.9999	0.4
Bx	21	■	■	■	□	■	□	■	■	■	■	■	□	■	■	■	■	2	1.4	1	0.6	0.8495	0.2
Bx	20	■	■	■	□	□	□	■	■	■	■	■	□	■	■	■	■	2	1.4	0	0.0	0.1799	-
Bx	10	■	□	■	□	■	□	■	□	■	■	■	□	■	□	■	■	1	0.7	2	1.2	> 0.9999	-
Bx	11	■	■	■	■	■	■	■	□	■	■	■	□	■	■	■	■	0	0.0	1	0.6	> 0.9999	-
Bx	12	■	□	■	■	■	□	■	■	■	■	■	■	■	■	■	■	1	0.7	1	0.6	> 0.9999	0.9
Bx	13	■	■	■	□	■	■	■	□	■	■	■	■	■	■	■	■	0	0.0	1	0.6	> 0.9999	-
Bx	91	■	■	□	■	■	■	■	■	■	■	■	□	■	■	■	■	0	0.0	2	1.2	> 0.9999	-
Bx	56	■	□	■	■	■	■	■	■	■	■	■	■	■	■	■	■	3	2.2	0	0.0	0.07995	-
Bx	112	■	■	□	□	■	■	■	■	■	■	■	□	■	■	■	■	1	0.7	0	0.0	0.4031	-
Bx	74	□	■	□	■	■	□	□	■	■	□	■	■	■	■	□	■	1	0.7	0	0.0	0.4031	-
Bx	89	■	■	□	□	■	□	■	□	■	■	■	□	■	□	■	■	1	0.7	0	0.0	0.4031	-
Bx	77	□	■	□	■	■	□	■	■	■	■	■	□	■	■	■	■	2	1.4	0	0.0	0.1799	-
Bx	101	□	■	□	■	■	■	□	■	■	□	■	■	■	■	■	■	1	0.7	0	0.0	0.4031	-
Bx	18	■	■	■	■	□	□	■	■	■	■	■	■	■	■	■	■	1	0.7	1	0.6	> 0.9999	0.7
Bx	32	■	□	■	□	□	□	■	■	■	■	■	□	■	■	■	■	1	0.7	0	0.0	0.4031	-
Bx	51	■	■	■	□	□	■	■	□	■	■	■	□	■	■	■	■	0	0.0	1	0.6	> 0.9999	-
Bx	14	■	□	■	□	□	□	■	□	■	■	■	■	■	□	■	■	0	0.0	1	0.6	> 0.9999	-
Bx	228	■	■	□	□	■	□	■	■	■	■	■	□	■	■	■	■	0	0.0	1	0.6	> 0.9999	-

The presence of the *KIR* gene was represented by black boxes and the absence of the gene was indicated by white boxes. n: number of individuals; %: haplotype group frequency; p-value[1]: refers to analysis without gender and age adjustment; p-value[2]: refers to the logistic regression analysis adjusted for gender and age. ID genotype is assigned by the Allele Frequencies Net Database. The order of the genes is according to the genomic position (centromeric to telomeric).

CC BY

TABLE II

Frequency distribution of the *KIR* genes and KIR-human leukocyte antigen (HLA) class I in Zika virus (ZIKV) patients and controls by logistic regression with adjustment for age and sex

KIR genes	Patients (N= 139)	Controls (N= 170)	Unadjusted		Adjusted	
Inhibitory	n (%)	n (%)	OR (95% CI)	p-value	OR (95% IC)	p-value
2DL1	130 (93.5)	161 (94.7)	0.81 (0.31-2-12)	0.7	0.69 (0.24-1.96)	0.5
2DL2	78 (56.1)	90 (52.9)	1.12 (0.72-1.76)	0.6	1.47 (0.90-1.44)	0.13
2DL3	119 (85.6)	150 (88.2)	0.79 (0.41-1.55)	0.5	0.79 (0.38-1.64)	0.5
2DL5	74 (53.2)	89 (52.4)	1.04 (0.66-1.62)	0.9	0.90 (0.55-1.47)	0.7
3DL1	134 (96.4)	159 (93.5)	1.85 (0.66-6.01)	0.3	2.08 (0.67-7.21)	0.2
Activating						
2DS1	53 (38.1)	69 (40.6)	0.90 (0.57-1.43)	0.7	0.81 (0.49-1.33)	0.4
2DS2	79 (56.8)	90 (52.9)	1.17 (0.75-1.84)	0.5	1.55 (0.94-2.57)	0.088
2DS3	41 (29.5)	48 (28.2)	1.06 (0.65-1.74)	0.8	1.00 (0.58-1.72)	>0.9
2DS5	51 (36.7)	62 (36.5)	1.01 (0.63-1.61)	> 0.9	0.94 (0.57-1.57)	0.8
3DS1	51 (36.7)	66 (38.8)	0.91 (0.57-1.45)	0.7	0.81 (0.49-1.34)	0.4
2DS4	134 (96.4)	159 (93.5)	1.85 (0.66-6.01)	0.3	2.08 (0.67-7.21)	0.2
Pseudogene						
2DL4	139 (100)	170 (100)	-	-	-	-
3DL2	139 (100)	170 (100)	-	-	-	-
3DL3	139 (100)	170 (100)	-	-	-	-
3DP1	139 (100)	170 (100)	-	-	-	-
2DP1	133 (95.7)	161 (94.7)	1.24 (0.44-3.78)	0.7	0.93 (0.30-3.06)	>0.9
KIR-HLA class I						
2DL1-C2	88(63.3)	113 (66.5)	1.92 (0.40-13.7)	0.4	1.56 (0.29-11.8)	0.6
2DL2-C1	63 (45.3)	69 (40.6)	0.98 (0.44-2.21)	> 0.9	0.91 (0.38-2.22)	0.8
2DL3-C1	94 (67.6)	119 (70.0)	0.99 (0.25-4.09)	> 0.9	1.28 (0.27-6.96)	0.8
3DL2-A*03/A*11	43 (30.9)	43 (25.3)	1.32 (0.80-2.18)	0.3	1.22 (0.70-2.11)	0.5
3DL1-Bw4	106 (76.3)	136 (80.0)	0.93 (0.56-1.56)	0.8	0.87 (0.50-1.52)	0.6
2DS1-C2	36 (25.9)	52 (30.6)	0.89 (0.46-1.71)	0.7	0.80 (0.39-1.63)	0.5
2DS2-C1	60 (43.2)	71 (41.8)	1.16 (0.52-2.62)	0.7	1.10 (0.46-2.68)	0.8
2DS4-C*04	46 (33.1)	48 (28.2)	1.30 (0.50-3.63)	0.6	1.50 (0.52-4.48)	0.5

n: number of individuals; %: frequency; CI: confidence interval; OR: odds ratio.
